# The Predictive Paradigm in Perioperative Hemodynamic Management: The Role of Artificial Intelligence in Major Spine Surgery

**DOI:** 10.3390/jcm15176915

**Published:** 2026-09-07

**Authors:** Gianluigi Cosenza, Marco Fiore, Roberto Giurazza, Vincenzo Pota, Francesco Coppolino, Pasquale Sansone, Maria Caterina Pace

**Affiliations:** 1 Department of Women, Child and General and Specialized Surgery, University of Campania “Luigi Vanvitelli”, 80138 Naples, Italy; marco.fiore@unicampania.it (M.F.); roberto.giurazza@unicampania.it (R.G.); vincenzo.pota@unicampania.it (V.P.); francesco.coppolino@unicampania.it (F.C.); pasquale.sansone@unicampania.it (P.S.); mariacaterina.pace@unicampania.it (M.C.P.); 2 Department of Critical Care, AORN Ospedali dei Colli, Via Leonardo Bianchi, 80131 Naples, Italy

**Keywords:** artificial intelligence, machine learning, predictive anesthesia, hemodynamic management, major spine surgery, Hypotension Prediction Index (HPI)

## Abstract

**Background:** Major spine surgery carries an inherent risk of hemodynamic instability due to prone positioning, significant blood loss, and the strict necessity to maintain adequate spinal cord perfusion. Hemodynamic management mainly relies on a reactive approach, treating hypotension only after it occurs, which increases the risk of postoperative complications such as acute kidney injury and ischemic events. This narrative review evaluates the clinical impact, current evidence, and future perspectives of integrating Artificial Intelligence (AI) and Machine Learning (ML) algorithms into perioperative hemodynamic care. **Methods:** A comprehensive literature search was conducted through PubMed, EMBASE, and the Cochrane Library, spanning from inception to January 2026. The search strategy employed combinations of Medical Subject Headings terms and keywords related to “Artificial Intelligence,” “Machine Learning,” “Hypotension Prediction Index,” “hemodynamic monitoring,” and “major spine surgery.” Studies were selected based on their relevance to predictive hemodynamic algorithms, goal-directed fluid therapy (GDFT), and automated closed-loop systems within the perioperative setting of complex spinal interventions. **Results:** Five studies show that AI/ML tools can improve hemodynamic management in spine surgery: an hypotension prediction index (HPI)-guided algorithm reduced intraoperative hypotension during prone spinal fusion; a machine learning model accurately predicted massive blood loss in metastatic spinal disease; an AutoML framework linked intraoperative hypertension to worse neurological recovery after spinal cord injury (SCI); a case report showed HPI-guided goal-directed therapy enabled safe, transfusion-free major spine surgery; and topological network analysis identified a narrow optimal mean arterial pressure (MAP) range for neurological recovery after SCI. Collectively, these preliminary findings suggest a potential role for AI/ML in reducing hemodynamic instability and enabling more individualized perioperative management in spine surgery. Rather than converging on a single verdict, these five studies fall into three distinct evidentiary categories when appraised using a structured model-validation (V1–V4) and clinical-translation (T0–T4) framework applied within each category: a real-time monitoring technology (HPI) with a substantial extra-spinal evidence base but a limited spine-specific replication record; a single, externally validated but clinically unproven preoperative prediction model; and two retrospective, hypothesis-generating discovery frameworks that remain exploratory irrespective of surgical domain. **Conclusions:** The evidence identified does not support a single, unified statement about “AI/ML in spine surgery.” Instead, it points to three distinct situations that warrant separate research priorities: consolidating spine-specific replication of an otherwise mature monitoring technology (HPI); externally confirming the clinical utility, rather than only the discriminative accuracy, of a single preoperative prediction model; and prospectively testing the retrospectively derived targets generated by discovery-oriented analytic frameworks. Considered together, these findings should inform hypothesis-driven research design rather than a single implementation-readiness judgment.

## 1. Introduction

For the purposes of this review, artificial intelligence (AI) is defined as the broader field of computational systems capable of performing tasks that typically require human cognition, such as pattern recognition, prediction, and decision support, while machine learning (ML) refers to a specific subset of AI in which algorithms learn patterns directly from data to generate predictions or classifications without being explicitly programmed for each individual task. Throughout this review, the terms AI and ML are used according to these definitions, with ML-based tools discussed as a specific, data-driven implementation of the broader AI paradigm applied to perioperative hemodynamic monitoring.

Intraoperative hypotension (IOH) is considered a high-burden problem leading to acute kidney injury (AKI) [1,2], acute myocardial injury after surgery (AMI/MINS), acute hepatic injury [3] and death [4].

Major spine surgery presents several challenges for the anesthesiologist due to an impairment and rearrangement in the patient’s hemodynamics. Prone position can compromise cardiovascular physiology by reducing venous return through inferior vena cava compression and by increasing intrathoracic pressure secondary to decreased thoracic compliance, ultimately limiting left ventricular preload [5,6,7].

Beyond these systemic hemodynamic derangements, spine surgery poses a challenge that is largely distinctive among surgical specialties: the need to maintain an adequate mean arterial pressure (MAP) to preserve spinal cord perfusion, particularly during procedures involving instrumentation near the spinal cord or in the setting of acute spinal cord injury. Unlike most other vascular beds, the spinal cord has limited autoregulatory reserve under conditions of surgical manipulation and altered posture, such that sustained deviations from an optimal MAP range—whether hypotensive or hypertensive—may directly compromise neurological outcome. This specific vulnerability differentiates the hemodynamic priorities of spine surgery, and particularly of SCI surgery, from those of general surgical procedures, in which blood pressure targets are typically set to protect systemic organ perfusion rather than spinal cord perfusion specifically.

In addition, stress response and systemic inflammatory response syndrome (SIRS) may further destabilize hemodynamics by inducing systemic vasodilation, thereby increasing the risk of IOH [8,9]. These complex physiological interactions highlight the importance of accurate and continuous hemodynamic monitoring throughout the perioperative period. Furthermore, prolonged duration and potentially significant blood loss require strict monitoring.

Current hemodynamic monitoring strategies range from conventional non-invasive methods, such as intermittent non-invasive blood pressure (NIBP) measurement and electrocardiography (EKG), to advanced invasive and minimally invasive technologies. In patients undergoing complex spine procedures, continuous arterial blood pressure monitoring through an arterial catheter is frequently employed, allowing real-time assessment of blood pressure and facilitating serial blood gas analyses [10]. More advanced monitoring systems provide dynamic parameters of fluid responsiveness, including stroke volume variation (SVV), pulse pressure variation (PPV), cardiac output (CO), and cardiac index (CI), which can support individualized fluid therapy within a goal-directed therapy (GDT) approach [11] aiming to optimize preload, CO, and tissue perfusion while avoiding unnecessary fluid administration [12]. Several studies have demonstrated that GDT may reduce postoperative complications and shorten hospital stay in high-risk surgical populations, although evidence specific to major spine surgery remains heterogeneous [13,14].

During major spine surgery, prolonged operative times, prone positioning, significant blood loss, and rapid hemodynamic changes can make timely clinical decision-making challenging [15]. Therefore, artificial intelligence (AI) and machine learning (ML) algorithms can improve perioperative hemodynamic monitoring by facilitating the real-time analysis of large volumes of complex physiological data, enabling the early prediction of hemodynamic instability, such as IOH, before conventional monitoring detects clinically relevant changes [16]. By supporting individualized fluid and vasopressor management, AI may enhance the effectiveness of GDT and contribute to more personalized perioperative care. Although evidence in spine surgery is still limited, the promising results reported in other high-risk surgical settings suggest that AI could become a valuable adjunct to conventional hemodynamic monitoring [17,18,19].

Despite growing interest in the application of AI to perioperative medicine, evidence supporting its use for hemodynamic monitoring during major spine surgery remains limited. This narrative review aims to critically examine the available literature on AI-based approaches for perioperative hemodynamic monitoring in this setting, with particular emphasis on their current clinical applications, potential impact on patient management, existing limitations and knowledge gaps and discuss future research directions to facilitate the integration of AI into perioperative hemodynamic management in this high-risk surgical population.

## 2. Materials and Methods

This narrative review was informed by the general principles of the Scale for the Assessment of Narrative Review Articles (SANRA) [20] (Table S1), rather than by the PRISMA or PRISMA-ScR reporting standards developed specifically for systematic and scoping reviews. Although a structured, multi-database search strategy was applied to identify relevant literature in a systematic and reproducible manner, this review does not claim exhaustive literature coverage, does not report a formal, study-level risk-of-bias assessment, and does not include a PRISMA-type identification–screening–eligibility–inclusion flow diagram; these are inherent features of the narrative review format adopted here rather than omissions from a systematic review methodology.

A comprehensive literature search was conducted in PubMed, EMBASE, and the Cochrane Library from database inception to January 2026. The search strategy combined Medical Subject Headings (MeSH) and free-text terms related to *artificial intelligence*, *machine learning*, *hemodynamic monitoring*, *Hypotension Prediction Index*, and *major spine surgery*. Papers published in English were screened to identify studies addressing AI-based approaches to perioperative hemodynamic monitoring. Attention was given to predictive algorithms, GDT, automated closed-loop systems, and other AI-driven technologies with potential applications in major spine surgery.

Studies were eligible for inclusion if they specifically evaluated an artificial intelligence (AI)—or machine learning (ML)-based tool applied to intraoperative hemodynamic monitoring, prediction, or management in adult patients undergoing major spine surgery or surgery for spinal cord injury (SCI)—and reported at least one hemodynamic, hemorrhagic, or outcome-related endpoint. Randomized controlled trials, case reports, cohort studies, and model-development studies were all considered eligible, provided that an AI/ML component was explicitly described. Studies were excluded if they involved non-spinal surgical populations, did not incorporate an AI/ML-based tool (e.g., conventional goal-directed therapy without a predictive algorithm), addressed AI/ML applications unrelated to hemodynamic management (e.g., prediction of postoperative delirium, intraoperative neuromonitoring, or postoperative pain), were conference abstracts, editorials, or narrative commentaries without original data, or were not available in English; such related but hemodynamically distinct AI/ML applications in spine surgery were considered outside the scope of this review and may warrant separate, dedicated evaluation. Titles and abstracts were screened for relevance, followed by full-text review of potentially eligible articles; screening was performed by a single reviewer (G.C.), and no disagreements requiring adjudication arose given this single-reviewer process. Given the narrow intersection between AI/ML methodology and spine-surgery-specific hemodynamic management, and consistent with the exploratory, narrative nature of this review, the search was designed to be broad and hypothesis-generating rather than exhaustive; consequently, only five studies meeting all eligibility criteria were identified, reflecting the current scarcity of dedicated evidence in this specific surgical field rather than an overly restrictive selection process.

To provide a structured, descriptive assessment of the maturity of the available evidence, the included studies were mapped across two complementary but distinct domains: model validation maturity and clinical translation maturity. This framework was not intended as a formal quality score or risk-of-bias assessment, but rather to describe how far each model or AI-based technology had progressed from analytical development to clinical evaluation.

Model validation maturity was classified as V1, development or exploratory analysis only; V2, internal validation using resampling, cross-validation, bootstrapping, or data splitting; V3, temporal validation in a later cohort from the same institution or healthcare system; and V4, independent external validation in a different institution, geographic setting, or healthcare system. Studies evaluating the clinical application of an already developed proprietary algorithm, without reassessing its predictive performance, were classified as not assessable for this domain.

Clinical translation maturity was classified as T0, analytical use without a specified clinical action; T1, specification of a potentially actionable risk threshold, hemodynamic target, or model-informed clinical strategy without prospective implementation; T2, prospective evaluation in which model outputs did not influence patient management; T3, prospective model-guided clinical implementation or feasibility assessment; and T4, comparative impact evaluation examining whether model-guided care affected clinical decisions, physiological endpoints, resource use, or patient outcomes.

Clinical actionability was additionally recorded through the item “Evidence of model-driven clinical action or decision-threshold specification,” defined as the presence of an explicit risk threshold, therapeutic target, recommended intervention, or documented modification of clinical management linked to the model output.

This maturity framework was developed by the authors specifically for this narrative review, to provide a structured, descriptive overview of evidence maturity in a field where recognized appraisal tools remain limited in scope. It is conceptually related to, but distinct from, established frameworks such as TRIPOD + AI and PROBAST, which focus on transparent reporting and risk-of-bias assessment for individual prediction models, and the IDEAL-D framework, which describes stages of surgical device and technology innovation; unlike these tools, the present framework was designed to jointly capture both analytical validation and clinical implementation status for AI/ML-based hemodynamic technologies within a single narrative synthesis. It has not been externally validated, and formal inter-rater reliability was not assessed; classification of each study against the framework was performed by a single reviewer (M.F.). It should therefore be interpreted as a descriptive, hypothesis-generating tool rather than a validated appraisal instrument, and the results derived from it should be interpreted with corresponding caution.

## 3. Results

Five studies (Table 1) that evaluated the application of AI and ML tools for hemodynamic monitoring and outcome prediction in the context of spine surgery were identified, spanning from intraoperative blood pressure management, to patient blood management, and prognostic modeling. These five studies do not merely differ in design; they represent three categorically distinct tools answering fundamentally different clinical questions: a real-time, waveform-based hemodynamic monitoring technology (the Hypotension Prediction Index, HPI, Edwards Lifesciences Corporation, Irvine, CA, USA), a preoperative outcome-prediction model, and two retrospective discovery frameworks that identify associations or candidate target ranges without testing a prospective intervention (Table 1). Because these categories are not mechanistically comparable, they are presented and synthesized separately below, by tool type, rather than pooled into a single narrative about “AI/ML in spine surgery,” as detailed further in the Discussion.

**Real-time hemodynamic monitoring: the Hypotension Prediction Index (HPI).** Regarding IOH prevention, Pilakouta Depaskouale et al. [21] conducted a single-center, single-blind randomized controlled trial in 85 adults undergoing prolonged prone-position spine fusion surgery, randomized 1:1 to an active Hypotension Prediction Index (HPI)-guided management strategy or to standard reactive care with HPI readings blinded to the clinical team. Seventy-seven patients (39 in the intervention group) were included in the final analysis, as 8 patients were excluded for technical reasons. Contrary to the study hypothesis, no statistically significant difference was found between groups in the primary outcome—the time-weighted average (TWA) of IOH—and postoperative complications did not differ either. This null result for the primary endpoint contrasts with the broader HPI literature, in which subsequent systematic reviews and meta-analyses across other surgical settings have more consistently reported reductions in the TWA and duration of IOH with HPI guidance, generally without a corresponding reduction in postoperative complications such as AKI. Complementing this trial-level evidence at the level of an individual clinical application, Denn et al. [22] described a case in which HPI-guided, fluid-restrictive GDT was used to manage an elderly Jehovah’s Witness patient undergoing major spinal surgery without the possibility of blood product transfusion, allowing clinicians to anticipate and preempt hypotensive episodes while avoiding both hypovolemia and hypervolemia. Taken together, these two studies indicate that HPI is a comparatively mature monitoring technology—with a substantial evidence base of randomized trials and meta-analyses accumulated outside spine surgery—for which spine-specific replication currently consists of a single, statistically null randomized trial and a single case report; the limiting factor here is a spine-specific replication gap rather than immaturity of the underlying technology itself.

**Preoperative prediction of massive blood loss.** Addressing perioperative hemorrhagic risk from a predictive rather than a monitoring standpoint, Shi et al. [23] developed and validated a web-based AI model, based on an XGBoosting machine (XGBM) algorithm, to predict massive intraoperative blood loss in patients undergoing decompressive surgery for metastatic spinal disease. The model demonstrated good discriminative performance and was subsequently deployed as a freely accessible online calculator, illustrating how ML-based risk-stratification tools can be translated into practical, bedside-usable decision aids for anticipating transfusion needs and guiding preoperative planning. Unlike HPI, this model has no substantial evidence base outside its original clinical niche; its position on the maturity scale therefore reflects genuine early-stage development of the tool itself, rather than merely a lack of spine-specific replication.

**Retrospective discovery frameworks: AutoML and topological network analysis.** Beyond real-time titration and preoperative prediction, two further studies applied retrospective, hypothesis-generating discovery frameworks to spinal cord injury (SCI) cohorts. Chou et al. [24] developed an expert-augmented AutoML framework applied to intraoperative hemodynamic data from the TRACK-SCI cohort, which improved the reproducibility and clinical interpretability of predictive models relative to conventional AutoML pipelines and identified a previously underappreciated detrimental association between intraoperative hypertension and neurological recovery. In a related multicenter effort, Torres-Espín et al. [25] applied topological network analysis—an unsupervised machine intelligence technique combining nonlinear dimensionality reduction and persistent homology—to continuous mean arterial pressure (MAP) and heart rate recordings from 118 patients undergoing acute SCI surgery across two Level 1 trauma centers, identifying a narrow optimal intraoperative MAP range (approximately 76 to 104–117 mmHg) associated with the highest likelihood of neurological recovery. Neither study tested a prospective intervention; both instead generated candidate associations or target ranges from observational data, reinforcing the concept of a hemodynamic “sweet spot” rather than a simple threshold-based rule. Because association- and target-discovery methodologies of this kind are inherently exploratory wherever they are applied, these two studies occupy an early stage on the maturity scale for reasons distinct from those affecting HPI or the Shi et al. model: the limiting factor here is the exploratory nature of the analytic method itself, not merely a scarcity of spine-specific data.

Collectively, these five studies do not converge on a single verdict about “AI/ML in spine surgery.” Rather, they illustrate three distinct evidentiary situations, each requiring its own research pathway: (i) a monitoring technology (HPI) with substantial extra-spinal evidence but a spine-specific replication gap illustrated by a single null randomized trial (Pilakouta Depaskouale et al. [21]) and a single case report (Denn et al. [22]); (ii) a single, internally and externally validated but clinically unproven preoperative prediction model (Shi et al. [23]); and (iii) two retrospective, hypothesis-generating discovery frameworks that remain exploratory irrespective of surgical domain (Chou et al. [24]; Torres-Espín et al. [25]). These three situations are presented and interpreted separately in the Section 4, rather than pooled into a single narrative-maturity conclusion.

**Table 1:** Included studies in the narrative synthesis.

Authors	Year	Study Type	N. of Patients	Surgical Population	AI Model/Technology	Outcome	Results	Model Validation Maturity	Clinical Translation Maturity
Pilakouta Depaskouale et al. [21]	2025	**Category A—Real-time monitoring (HPI)** Single-center clinical trial	77	Posterior spinal fusion surgery	Hypotension Prediction Index (HPI) software	Time Weighted Average (TWA) of IOH and comparison of postoperative complications.	No statistically significant difference between groups in TWA of IOH (primary outcome, 77 patients analyzed); postoperative complications did not differ.	NA *	T4
Shi et al. [23]	2024	**Category B—Preoperative prediction** Observational cohort study	276	Decompressive surgery	XGBoosting machine (XGBM), K-Nearest Neighbor (KNN), Support Vector Machine (SVM), Decision Tree (DT), and Random Forest (RF).	Intraoperative blood loss.	The XGBM model obtained the best predictive performance among the evaluated models.	V2	T1
Chou et al. [24]	2022	**Category C—Retrospective discovery framework** Retrospective cohort study using automated machine learning.	74	Spinal cord injury (SCI) surgery	AutoML	Optimize hemodynamics in the intraoperative and identify a relation with hypertension and patient outcome.	AutoML-derived models supported an optimal intraoperative hemodynamic range and identified a detrimental relationship between intraoperative hypertension and patient outcome.	V2	T1
Denn et al. [22]	2022	**Category A—Real-time monitoring (HPI)** Case report	1	Spinal fusion surgery	HPI software	Reduce the risk of IOH and transfusion in a Jehovah’s Witness patient.	The HPI-based treatment algorithm is useful during complex anesthesia and the perioperative period.	NA *	T3
Torres-Espin et al. [25]	2021	**Category C—Retrospective discovery framework** Retrospective observational study	118	SCI surgery	Topological network analysis (persistent homology–based machine intelligence)	Identify the optimal MAP target to enhance surgical recovery after SCI surgery.	The optimal MAP target for optimizing surgical recovery was 76–(104–117) mmHg.	V1	T1

* Model validation maturity was not assessable for studies evaluating an already-developed, proprietary algorithm (Hypotension Prediction Index software) without reassessing its underlying predictive performance in the present study.

## 4. Discussion

The evidence synthesized in this narrative review remains preliminary, both in volume and in methodological maturity. More importantly, the five core studies identified do not merely differ in study design (one randomized controlled trial [21], one single-patient case report [24], and three model-development, prognostic-modeling, or exploratory network-analysis studies [22,23,25]); they represent three categorically distinct tools addressing fundamentally different clinical questions, with different starting points on any evidence-maturity scale. Sample sizes were generally modest—ranging from a single case [24] to cohorts of fewer than 300–500 patients [21,22,23,25]—and most studies originated from single centers or a limited number of collaborating institutions, raising concerns about generalizability across diverse surgical populations, anesthetic protocols, and monitoring equipment. Because of this categorical, rather than merely design-level, heterogeneity, we do not synthesize these studies into a single narrative-maturity conclusion; instead, findings are considered separately by tool type below, and the model-validation (V1–V4) and clinical-translation (T0–T4) framework introduced in the Methods is applied within, rather than pooled across, each category. This distinction matters in particular for the framework’s V1–V4 axis, which describes the analytic validation status of a given model as assessed in this narrative synthesis; it is not a measure of the overall maturity of the underlying technology platform in other clinical domains, a distinction that is especially relevant to the Hypotension Prediction Index (HPI), discussed below.

**Real-time hemodynamic monitoring: the Hypotension Prediction Index (HPI).** Broader meta-analytic evidence on HPI-guided hemodynamic management, drawn predominantly from non-cardiac and abdominal surgery rather than spine surgery specifically, offers some indirect reassurance regarding the underlying technology: pooled analyses of randomized trials have consistently shown reductions in the TWA and duration of IOH with HPI guidance, without a corresponding reduction in postoperative complications such as AKI [18,26,27,28,29]. This pattern—a robust physiological signal alongside inconsistent clinical-outcome benefit—mirrors what has been reported for the prone spine surgery trial included in this review [21], and underscores that a reduction in intermediate hemodynamic endpoints does not yet constitute proof of improved patient-centered outcomes, in spine surgery or elsewhere. Similarly, a large multicenter randomized trial in abdominal surgery has provided a template for the type of adequately powered, multi-institutional design that spine surgery research in this area still largely lacks [18]. One plausible explanation for the recurring dissociation between improved intermediate hemodynamic metrics and unchanged postoperative outcomes relates to the physician-mediated nature of the intervention itself. Predictive software such as the HPI generates an early warning of impending hypotension, but the decision of whether, when, and how to intervene—through fluid administration, vasopressor titration, or a change in surgical or anesthetic technique—remains entirely at the discretion of the treating clinician. Variability in individual interpretation of predictive alerts, differing thresholds for intervention, and inconsistent adherence to any predefined hemodynamic protocol may therefore attenuate the clinical impact of an otherwise accurate prediction, irrespective of the underlying algorithm’s performance. This human-in-the-loop dependency represents an important, and often underappreciated, mediating factor between AI-based prediction and downstream patient outcomes, and should be considered when interpreting the modest or absent effect of HPI guidance on postoperative complications across the reviewed literature. Taken together, the case for HPI is best summarized as follows: the technology itself is comparatively mature, but spine-specific replication is limited to a single, statistically null randomized trial [21] and a single case report [24], and greater translational maturity within this review’s T0–T4 framework (reflecting the comparative evaluation performed by Pilakouta Depaskouale et al.) does not equate to demonstrated clinical benefit, since that same trial failed to demonstrate a consistent improvement in its primary hypotension endpoint or postoperative complications.

**Preoperative prediction of massive blood loss.** The evidentiary situation for preoperative outcome prediction is different in kind. The blood-loss prediction model developed by Shi et al. underwent internal validation at the time of publication [22], and a subsequent multi-institutional external validation study involving 880 patients has since been reported, lending some additional support to its generalizability [29]. However, neither predictive accuracy nor the availability of a web-based calculator demonstrates that use of the model improves transfusion planning or patient outcomes, and—unlike HPI—there is no substantial body of evidence for this specific model outside its original spinal-surgery niche to draw on. Its early position on the clinical-translation axis (T0–T1) therefore reflects genuine early-stage development of the tool itself, not merely a lack of replication within a mature field.

**Retrospective discovery frameworks: AutoML and topological network analysis.** The evidentiary situation for the two retrospective discovery frameworks—Chou et al.’s expert-augmented AutoML analysis and Torres-Espín et al.’s topological network analysis—is different again. Both generated potentially actionable hemodynamic ranges or associations from retrospective data, but neither has been prospectively validated as a treatment threshold, and neither tested a prospective intervention. Association- and target-discovery methodologies of this kind are inherently exploratory (model-validation stage V1–V2) wherever they are applied, in any surgical domain; their early-stage classification in this review therefore reflects the intrinsic nature of the analytic approach rather than a spine-specific evidence gap, and should not be read as directly comparable to the replication gap affecting HPI or the single-model gap affecting the Shi et al. tool.

Several additional, cross-cutting limitations warrant emphasis irrespective of tool category. First, none of the core studies reported long-term functional or patient-centered outcomes beyond the immediate perioperative period, with the partial exception of the spinal cord injury cohorts, in which neurological recovery was assessed as a downstream endpoint [23,25]. Second, publication and reporting bias cannot be excluded, since studies demonstrating a positive or clinically intuitive association between AI-guided monitoring and favorable hemodynamic outcomes may be preferentially published over neutral or negative findings. In addition, this review was restricted to English-language, peer-reviewed publications indexed in the searched databases; conference proceedings, theses, and other gray literature sources were not systematically searched. Given the rapid pace of development in AI/ML methodology, relevant preliminary findings may currently exist only in conference abstracts or non-indexed sources, and their exclusion may further limit the completeness of the evidence base presented here. Finally, transparent and standardized reporting of AI-based prediction models remains inconsistent across the field, a concern that has prompted the recent development of dedicated reporting guidelines for AI studies in surgery [30].

**Barriers to clinical implementation.** Beyond the methodological limitations discussed above, several practical barriers may hinder the translation of AI/ML-based hemodynamic monitoring into routine clinical practice in spine surgery, and are likely to apply differently across the three tool categories discussed here. These include the acquisition and maintenance costs of proprietary sensors and software platforms, which may limit adoption outside well-resourced centers; the need for dedicated training of anesthesia and nursing staff to correctly interpret and act upon algorithm-generated alerts; technical challenges in achieving interoperability between AI/ML platforms and the heterogeneous array of anesthesia machines, monitors, and electronic health record systems currently in clinical use; the risk of alarm fatigue and consequent alert desensitization among clinical staff exposed to frequent false-positive predictions, which may paradoxically reduce responsiveness to genuine hemodynamic events; the medicolegal implications of clinical decisions made in response to, or in disagreement with, AI-generated recommendations, an area in which regulatory and liability frameworks remain largely undefined; and the computational infrastructure, data storage, and cybersecurity requirements associated with continuous, high-frequency physiological data processing. These technical and human factors should be addressed alongside efficacy considerations in future studies evaluating the real-world implementation of AI/ML-based hemodynamic monitoring in spine surgery.

Despite these caveats, the trajectory of this evidence is noteworthy when viewed through the lens of precision medicine, albeit in three distinct ways rather than one. Unlike traditional, population-derived hemodynamic thresholds (e.g., a fixed mean arterial pressure cutoff of 65 mmHg), the AI/ML approaches reviewed here move toward individualized, data-driven targets—whether through predictive, pre-emptive titration of vasopressor and fluid therapy [21,24], through the identification of patient-specific optimal blood pressure ranges derived from large, real-world physiological datasets [23,25], or through preoperative risk stratification tools that can be deployed at the bedside [22]. This shift from reactive, threshold-based management to proactive, personalized decision support is conceptually well aligned with the broader precision-medicine paradigm, in which therapeutic strategies are tailored to the physiological trajectory of the individual patient rather than to population averages. In the technically demanding context of spine surgery—characterized by prolonged operative times, prone positioning, extensive blood loss, and, in the case of spinal cord injury, a narrow therapeutic window for neuroprotection—such individualized approaches may offer meaningful advantages over conventional monitoring paradigms, provided that the category-specific evidence gaps identified above are addressed first.

Taken together, the current evidence should be interpreted as hypothesis-generating rather than practice-defining, and this applies differently to each of the three categories discussed. For HPI, larger, adequately powered, multicenter randomized trials with standardized hemodynamic endpoints and long-term functional outcomes, conducted specifically in spine surgery, are needed to confirm whether the technology’s extra-spinal track record translates into this setting. For the Shi et al. prediction model, further external validation across diverse populations and healthcare settings, together with outcome-level (rather than purely discriminative) evaluation, is required. For the AutoML and topological-network-analysis discovery frameworks, prospective testing of the retrospectively derived hemodynamic targets is the necessary next step before these associations can be considered candidate treatment thresholds. Transparent reporting according to emerging AI-specific methodological guidelines [30] will also be essential across all three categories. We therefore do not regard the recurring signal across these disparate, low-to-moderate certainty study designs as evidence of a single, coherent, and increasingly mature field of “AI/ML in spine surgery”; rather, it justifies three separate, category-specific programs of further investigation, none of which, on its own or collectively, establishes artificial intelligence as ready for clinical adoption in the perioperative management of spine surgery.

## 5. Conclusions

This narrative review does not support a single, unified statement about “AI/ML in spine surgery.” The five studies identified represent three categorically distinct tools—a real-time hemodynamic monitoring technology, a preoperative outcome-prediction model, and two retrospective discovery frameworks—each occupying a different position on the model-validation and clinical-translation axes for different, category-specific reasons.

For the Hypotension Prediction Index, the underlying monitoring technology is comparatively mature, with a substantial evidence base accumulated outside spine surgery; the limiting factor is a spine-specific replication gap, illustrated by a single, statistically null randomized trial and a single case report identified in this review. For the preoperative blood-loss prediction model of Shi et al., internal and external validation have been achieved, but clinical utility beyond discriminative accuracy remains unproven, and no comparable model exists for direct context. For the AutoML and topological-network-analysis discovery frameworks of Chou et al. and Torres-Espín et al., the exploratory, hypothesis-generating nature of the underlying analytic approach is the limiting factor, irrespective of surgical domain.

Because it was not prospectively registered and did not follow a systematic or scoping review methodology, and because the underlying evidence base for each category remains small and preliminary, this review cannot, and should not, be used to support the clinical implementation of AI/ML-based hemodynamic monitoring in major spine surgery at this time, for any of the three categories discussed. Rather than a single implementation-readiness judgment, we propose three separate, category-specific research priorities: prospectively registered, adequately powered spine-specific trials to close the HPI replication gap; external, outcome-level validation of preoperative prediction models beyond Shi et al.’s; and prospective testing of the hemodynamic targets and associations generated by retrospective discovery frameworks. Findings arising from such category-specific studies, rather than the hypothesis-generating evidence summarized here, should inform any future clinical practice recommendations in this field.

## Data Availability

The corresponding author can provide databases and literature screening upon valid request.

## Supplementary Materials

The following supporting information can be downloaded at: https://www.mdpi.com/article/10.3390/jcm15176915/s1, Table S1: SANRA—Scale for the Assessment of Narrative Review Articles.

## Abbreviations

The following abbreviations are used in this manuscript:

AI	Artificial intelligence
ML	Machine learning
GDFT	Goal-directed fluid therapy
SCI	Spinal cord injury
MAP	Median arterial pressure
HPI	Hypotension predictive index
IOH	Intraoperative hypotension
AKI	Acute kidney injury
PPV	Pulse pressure variation
CO	Cardiac output
CI	Cardiac index
XGBM	XGBoosting machine
