# Supplementary material for: The Predictive Paradigm in Perioperative Hemodynamic Management: The Role of Artificial Intelligence in Major Spine Surgery
Source: J Clin Med. 2026 Sep 7;15(17):6915. doi: 10.3390/jcm15176915 (PMC13565961; doi:10.3390/jcm15176915)
Supplement: Supplementary file 1 [file jcm-15-06915-s001.zip › jcm-4496893-supplementary.pdf]

Table S1

SANRA — Scale for the Assessment of Narrative Review Articles

Manuscript: "The Predictive Paradigm in Perioperative Hemodynamic Management: The Role of Artificial Intelligence in Major Spine Surgery"

Authors: G. Cosenza, M. Fiore, R. Giurazza, V. Pota, F. Coppolino, P. Sansone, M.C. Pace

Assessed version: Revised_version.docx (post peer-review revision, incorporating Reviewer 1–3 comments)

Instrument reference: Baethge C, Goldbeck-Wood S, Mertens S. SANRA—a scale for the quality assessment of narrative review articles. Res Integr Peer Rev. 2019;4:5. doi:10.1186/s41073-019-0064-8.

Background items (not scored)

Background item (not scored)	Response	Justification
1. Is the topic relevant, and is the article suitable for this journal (i.e., in line with the journal's scope and readership)?	**Yes**	The manuscript addresses AI/ML-based hemodynamic monitoring and prediction in major spine surgery — a technically demanding, high-morbidity perioperative setting — which is directly relevant to the clinical and translational readership of the Journal of Clinical Medicine (anesthesiology, spine surgery, digital/precision medicine).
2. Are the aims of the article accurately reflected in its title, abstract, and/or keywords?	**Yes**	Title ("The Predictive Paradigm in Perioperative Hemodynamic Management: The Role of Artificial Intelligence in Major Spine Surgery"), Abstract (Background/Methods/Results/Conclusions structure), and Keywords (Artificial Intelligence; Machine Learning; Predictive Anesthesia; Hemodynamic Management; Major Spine Surgery; Hypotension Prediction Index) are internally consistent and accurately describe the review's scope and focus.

Scored items (0 = low standard, 1 = moderate standard, 2 = high standard)

SANRA item (scored 0–2)	Score	Justification (with manuscript location)
1. Justification of the article's importance for the readership — Does the article state why the topic is of importance to the readership?	**2**	The Introduction explicitly establishes the clinical burden of intraoperative hypotension (IOH) — linking it to AKI, myocardial injury, hepatic injury, and death — and identifies the specific gap addressed: hemodynamic management in spine surgery "mainly relies on a reactive approach," with AI/ML proposed as a way to shift toward proactive management. The unique vulnerability of spinal cord perfusion to MAP deviations (Introduction, p. 2–3) further sharpens the rationale specific to this surgical population.
2. Statement of concrete aims or formulation of questions — Does the article contain a clear statement of aims or research questions?	**2**	The Introduction ends with an explicit aim statement: "This narrative review aims to critically examine the available literature on AI-based approaches for perioperative hemodynamic monitoring in this setting,

		with particular emphasis on their current clinical applications, potential impact on patient management, existing limitations and knowledge gaps, and discuss future research directions..." (p. 3).
3. Description of the literature search — Does the article describe when, where (which databases), and how the literature search was conducted?	**1**	The Methods section specifies the databases searched (PubMed, EMBASE, Cochrane Library), the search period (inception to January 2026), and the MeSH/free-text terms used (p. 4). Explicit inclusion/exclusion criteria and single-reviewer screening (G.C.) are also reported. However, the search is not fully systematic: no exact Boolean search strings are provided, no record counts at each screening stage are reported, no PRISMA-type flow diagram is included, and screening/eligibility assessment was performed by a single reviewer without independent duplicate screening. The manuscript transparently acknowledges these as inherent features of the narrative (SANRA-informed) rather than systematic format (p. 4), which is good practice, but the search description itself remains only partially complete by SANRA's standard.
4. Referencing — Is referencing adequate, i.e., are claims/statements in the text supported by appropriate and traceable citations?	**2**	The manuscript cites 30 references, predominantly primary studies and systematic reviews/meta-analyses from 2019–2026, with claims consistently and specifically attributed (e.g., trial-specific results are cited to the corresponding primary study; broader HPI literature claims are cited to multiple meta-analyses, [18],[25]–[27]). Two duplicate reference-list entries identified during peer review (previously [18]/[25] and [23]/[31]) were corrected and the reference list renumbered consistently through [1]–[29], with a further reference ([30], Notaro et al. 2026) subsequently added by the authors to support a new comparator statement in the Conclusions.
5. Scientific reasoning — Does the article reflect the state of scientific knowledge (i.e., current evidence hierarchies) and present evidence-based reasoning, including an appraisal of the quality of the evidence discussed?	**2**	The Discussion explicitly stratifies the five included studies by design and evidentiary weight (one RCT, one case report, three model-development/retrospective studies; p. 10), introduces a bespoke model-validation (V1–V4) / clinical-translation (T0–T4) maturity framework to appraise evidence maturity independently of study design (p. 5, 12–13), and explicitly cautions that "prospective clinical evaluation should not be interpreted as synonymous with model validation, and external validation should not be interpreted as evidence of clinical effectiveness" (p. 12). The manuscript also identifies and corrects an internal inconsistency (the Pilakouta Depaskouale et al. trial's non-significant primary endpoint) and offers a plausible mechanistic explanation (physician-mediated decision-making, p. 10–11) for the observed dissociation between hemodynamic and clinical outcomes, rather than overstating causal benefit.
6. Appropriate presentation of data — Where relevant, are data (e.g., tables,	**2**	Table 1 (p. 8–9) summarizes all five included studies in a structured format (authors, year, study type, N, surgical

figures) presented in a way that allows the reader to verify the authors' reasoning, and are the quality/limitations of the underlying evidence discussed?		population, AI model/technology, outcome, results, model-validation maturity, clinical-translation maturity), with an explanatory footnote clarifying the "NA" rating for proprietary-algorithm studies. Limitations of the underlying evidence (small samples, single-center origin, heterogeneity, inconsistent external validation, publication/gray-literature bias) are explicitly and separately discussed (p. 10–11). One minor weakness: the "Results" cell for Denn et al. is reported qualitatively ("HPI-based treatment algorithm is useful...") rather than with a comparably structured outcome statement to the other rows, slightly limiting like-for-like comparison across table rows.

Total SANRA score: 11 / 12 (sum of the six scored items; each item rated 0 = low standard, 1 = moderate standard, 2 = high standard, per Baethge et al., 2019).

Summary interpretation

This narrative review scores highly on SANRA, reflecting the extensive methodological transparency added during peer review: explicit inclusion/exclusion criteria, an author-declared narrative (rather than systematic) format aligned with SANRA principles, a bespoke evidence-maturity framework used to temper causal claims, and explicit, separate discussion of each study type's evidentiary weight. The principal remaining limitation, reflected in the score for Item 3, is that the literature search — while multi-database and criterion-based — was conducted by a single reviewer without independent duplicate screening or a formal record-flow accounting (e.g., a PRISMA-style diagram); the manuscript itself transparently acknowledges this as an inherent feature of its chosen narrative format rather than an omission.
